# A Clinical Lecture on Abscess of the Brain

**Published:** 1903-04-18

**Authors:** H. F. Mole

**Affiliations:** Assistant Surgeon and Surgeon-in-Charge of the Aural Department, Bristol Royal Infirmary


### Hospital Clinics and Medical Progress.

### A CLINICAL LECTURE ON ABSCESS OF THE BRAIN.

By H. F. Mole, F.R.C.S., Assistant Surgeon and Surgeon-in-Charge of the Aural Department, Bristol Royal Infirmary.

Abscesses may arise in the brain from various causes, but those in connection with, and arising as a complication of, chronic suppuration in the middle ear are far the most common and are those which will most likely call for operative treatment on the part of the surgeon. They are situated either in the temporo-sphenoidal lobe or the lateral lobe of the cerebellum. Other causes are foreign bodies, punctured wounds and compound fractures. The abscess in these cases will most likely be in the frontal or parietal regions. Disease of the bones of the nose will sometimes cause an abscess in the frontal lobe and abscesses may arise in any part of the brain as a result of pyæmia, but are perhaps most commonly found in the occipital region. In disease of the ear and nose the infection takes place either by direct extension, by the lymphatics, or along a thrombosed vein. In pyæmia the infection is carried by the blood stream, and in the injuries mentioned the septic material is carried directly into the brain at the time of the accident. We will now proceed to consider more fully the abscesses arising in connection with middle ear disease. When situated in the temporo sphenoidal lobe they will most commonly be found in the lower and back part, viz., in that part directly overlying the tegmen tympani and antrum. When in the cerebellum the front and lower part, viz., the area nearest the antrum and ear, will be the part affected. As it is often impossible to distinguish between an abscess in these two situations, it will be well to take together those symptoms which may be found as a result of either, endeavouring later on to mention the symptoms peculiar to each. We must first of all remember that the abscess may be absolutely latent, that is to say, it may be present for, at any rate, many months, and perhaps years, without giving rise to any symptoms at all. It may then suddenly burst into the ventricles or meninges, when death will rapidly supervene. More often having been more or less latent for a variable time, the abscess somewhat rapidly enlarges and then symptoms referable to increased intracranial tension, and pressure on important parts appear. Perhaps the first of these is headache, varying in degree, but often very severe, most marked on the affected side, and there is sometimes tenderness on percussion over the seat of the disease. At about the same time some alteration in the mental state is observed. It is impossible to attach too much importance to this most important symptom, for it is oftentimes almost the only one we have to go on to make our diagnosis. It is particularly difficult in hospital practice, for the patients come to us as absolute strangers. We do not know what their mental capacity has been or how dull and stupid they may usually be, and we have to accept an often indifferent account from the friends. Amongst the very many different manifestations of this altered mental state may be mentioned a slowness of speech and some drowsiness. A little time is taken to answer a question, and it is then answered very slowly. The patient may simply appear stupid or even neurotic. There is sometimes loss of memory for words and events and a sentence may be commenced and broken off in the middle from either a lapse of memory or a temporary aphasia. The drowsiness may increase so that the patient, when not being spoken to, may relapse into a continuous sleepy state. He is not, however, at this stage comatose or anything approaching it, and he can be roused by shaking him and speaking to him, and he appears to have the power of pulling himself together for a minute or two and will answer questions quite rationally, almost immediately afterwards relapsing into his former sleepy state. Yawning has not unfrequently been observed as a symptom at this time.

Vomiting may or may not be present. If it is, it is of value, but its absence does not assist us much. Very much the same may be said of optic neuritis; it is as often absent as present—in temporo-sphenoidal abscess probably more often absent, and certainly more common with meningitis, and probably with sinus thrombosis than with cerebral abscess. A certain amount of paralysis of the hemiplegic type may be present, and when sufficiently obvious to be certain of, is of great value in the diagnosis. Very often it is but slightly marked, and not always discernible. It may be limited to the face, the paralysis being of the supra-nuclear type, or there may be some slight weakness of the arm or leg, or of these with the face. There may be ptosis, usually on the same side as the abscess, or paralysis of other ocular muscles. The pupils may be irregular, but as a rule not until coma sets in. There is frequently constipation, and there may be involuntary passage of urine. Giddiness is not uncommon, and reeling or staggering gait is occasionally present. A fairly constant sign is a sub-normal temperature. An abnormally slow pulse and slow respiration are also frequently present. As the disease progresses the paralysis may become more marked and the drowsiness may pass into coma. Convulsions may supervene and the patient gradually expires. More often, however, the patient dies somewhat suddenly without this gradual supervention of coma, and the post-mortem often fails to show the reason why, for although it may be surmised that the abscess has ruptured into the ventricle or meninges before death the autopsy often shows it to be otherwise. Notwithstanding this long train of symptoms which *may* be present, it not infrequently happens that there is insufficient evidence to enable us to make a definite diagnosis. Having, however, formed the opinion that an abscess is present in the brain we may be unable to say whether it is in the temporo-sphenoidal lobe or in the cerebellum. The following points may assist in the differentiation. In a cerebellar abscess there are more likely to be optic neuritis and vomiting, the pain will be in the occipital region, there may be retraction of the head, and inco-ordination of the cerebellar type may be present. Any hemiparesis there may be will be on the *same* side as the abscess. Slow pulse and respiration are more likely to be present owing to direct pressure on the medulla. When in the temporo-sphenoidal lobe the hemiparesis will be on the opposite side, and if the left side of the brain be affected there may be an affection of speech.

In most of these cases referred to there is a history of ear discharge at some time or other. It may have been years before, or it may be present at the time, or have ceased with the onset of cerebral symptoms. In the absence of any such sign the diagnosis becomes increasingly difficult, and should there be discharge from both ears, and no localising signs to point to which side is affected, and knowing that the paralysis may be on the same side as the abscess when in the cerebellum and on the opposite side when in the temporo-sphenoidal lobe, and furthermore that a paralysis of the facial nerve may occur from the disease in the temporal bone, it will readily be understood what great difficulties the surgeon may have to surmount before he can make his diagnosis with sufficient accuracy to warrant his opening the skull. With regard to the latter point, a case recently came before the writer's notice in which there was facial paralysis of the peripheral type present with a temporo-sphenoidal abscess on the opposite side, there being no ear disease on the same side as the paralysis. The autopsy subsequently showed no other cause for this paralysis than the abscess. Whenever a patient presents himself with any of the symptoms mentioned above as possibly suggesting brain abscess it is one's imperative duty to carefully examine both ears, and one will often find discharge in the meatus, or old perforations in the drums, notwithstanding that the friends have asserted that there has never been any discharge from the ear. Acute abscesses of the brain hardly ever arise in connection with middle-ear disease. They are most often associated with some injury to the skull and brain. The symptoms are high temperature and rigors with headache and signs of cerebral compression rapidly supervening and becoming fatal within a short time. They simulate meningitis closely. As this lecture is intended to deal especially with abscesses arising in connection with middle-ear disease, we will now proceed with their treatment, just mentioning that if left alone they are all fatal. Surgical procedure in these cases has altered somewhat during the last few years, and instead of going direct for the abscess by trephining over a definite spot so many centimetres behind the external auditory meatus and so many more above or below Reid's base line, we now go direct for the original mischief and work from it to the abscess. The reason for this is that by opening the abscess only we leave the original disease untouched, we may overlook an extradural collection of pus, and it has already been mentioned that the abscess, whether in the temporo-sphenoidal lobe or in the cerebellum, is most often in that part nearest the ear and fairly often forms by direct extension from it. When the symptoms do not indicate in which part of the brain the abscess is located the temporo-sphenoidal lobe should always be explored first, as it will be found here two or three times more frequently than in the cerebellum. It does not much matter what skin incision is made, but one that answers very well is a curved one over the mastoid convex backwards. The flap with the periosteum and the pinna are reflected forwards, and the mastoid antrum opened, the usual landmarks (spine of Henle and suprameatal fossa) being used. The ridge of bone separating the antrum from the tympanum is removed throwing the antrum and tympanum into one cavity, and the parts are curetted. This, the "complete mastoid" operation is the first step. The roof of the attic and antrum is then chiselled away and a portion of the squamous part of the temporal bone above, making a good exposure of that part of the dura mater underlying the temporo-sphenoidal lobe. If there be an abscess present the dura will bulge and pulsation will be absent, the dura may have a normal appearance, or it may have a yellowish colour or be covered with flakes of lymph and there may be an abscess between it and the bone, which will have been opened during the operation. The bulging dura is now incised in the direction of the vessels and the brain explored with a grooved director, a trocar and canula or an exploring syringe. The pus may be very fœtid or it may be sweet. It may be considered advisable, especially if there are sloughy pieces of brain in the abscess to irrigate the cavity gently with normal saline solution. A good opening must be made, and perhaps the finger gently insinuated along the exploring trocar is as good as anything for this purpose, and allows digital examination of the cavity. A drainage tube should be inserted and stitched to the skin flap. The remainder of the bone cavity should be filled with gauze and so much of the skin incision as appears indicated sewn up. Should no abscess be found in the temporo-sphenoidal lobe the cerebellum must be explored. This may be done in several ways. In the first place the inner wall of the already opened antrum may be chiselled away in a direction inwards and backwards so as to open that part of the posterior fossa which lies between the vertical portion of the lateral sinus behind and the posterior semicircular canal in front. In this way that part of the cerebellum most adjacent to the ear is exposed, and it is here that the abscess will most probably be. This is a very small area to work in, and it might be safer to work back from the antrum until the anterior part of the sinus is exposed and then work down alongside this to the spot indicated. A second method is to chisel away from the antrum downwards and backwards until the sinus is exposed, to continue over it and to open the posterior fossa at a spot behind the vertical part and below the horizontal part of the sinus. A third method is to make an entirely new opening away from the first one, trephining the skull through its thin under surface behind and internal to the mastoid process. In both the second and third methods the skin wound must be enlarged by an incision backwards from the middle of the first flap, and the skin, periosteum, and muscles raised. The second method is probably the best one, as much more room can be obtained than in the first and one is not working at such a depth from the surface. Only abscess of the brain has been mentioned in these operations, but it will be seen that a very slight modification of the procedure will deal with all the intracranial complications of otitis media. The prognosis, even after the abscess is opened, is very grave, but the earlier it is done the better will this be, so it behoves the surgeon to make the diagnosis as early as possible, often alone on some of the mental symptoms suggested above, remembering that if he wait for "something more definite" his patient may slip from his grasp and the autopsy reveal to his chagrin a large abscess easily amenable to surgical treatment.

